# Excited-state symmetry breaking in 9,10-dicyanoanthracene-based quadrupolar molecules: the effect of donor–acceptor branch length†

**DOI:** 10.1039/d1cp02376d

**Published:** 2021-07-02

**Authors:** Zoltán Szakács, Florian Glöcklhofer, Felix Plasser, Eric Vauthey

**Affiliations:** Department of Physical Chemistry, University of Geneva 30 Quai Ernest Ansermet Geneva Switzerland eric.vauthey@unige.ch; Department of Chemistry and Centre for Processable Electronics, Imperial College London, Molecular Sciences Research Hub 80 Wood Lane London W12 0BZ UK; Department of Chemistry, Loughborough University Loughborough LE11 3TU UK

## Abstract

Excited-state symmetry breaking is investigated in a series of symmetric 9,10-dicyanoanthracenes linked to electron-donating groups on the 2 and 6 positions *via* different spacers, allowing for a tuning of the length of the donor–acceptor branches. The excited-state properties of these compounds are compared with their dipolar single-branch analogues. The changes in electronic structure upon their optical excitation are monitored by transient electronic spectroscopy in the visible and near-infrared regions as well as by transient vibrational spectroscopy in the mid-infrared. Our results reveal that, with the shortest branches, electronic excitation remains distributed almost symmetrically over the molecule even in polar environments. Upon increasing the donor–acceptor distance, excitation becomes unevenly distributed and, with the longest one, it fully localises on one branch in polar solvents. The influence of the branch length on the propensity of quadrupolar dyes to undergo excited-state symmetry breaking is rationalised in terms of the balance between interbranch coupling and solvation energy.

## Abstract 2

Excited-state symmetry breaking in quadrupolar molecules depends on the balance between inter-branch coupling and polar solvation energy.

## Graphical abstract

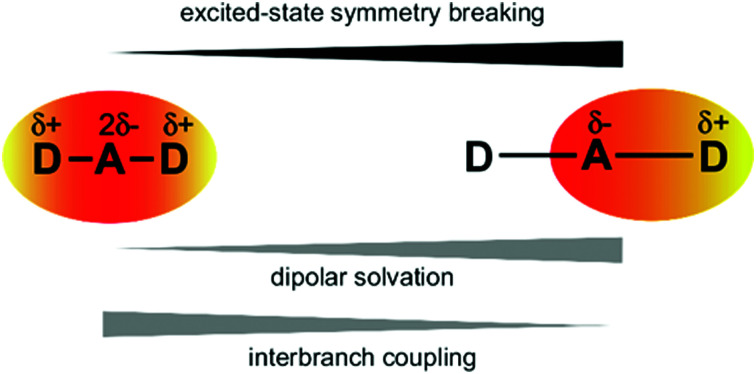

## 1 Introduction

**Fig. 1:**
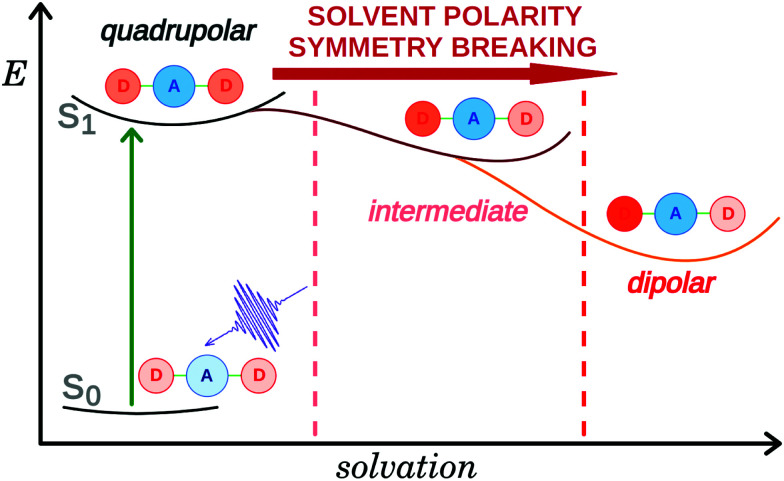
Schematic representation of excited-state symmetry breaking in a D–A–D molecule.

The interest for symmetric multi-branched electron donor–acceptor (DA) dyes has considerably increased over the past few years. These compounds are usually characterised by a large two-photon absorption cross-section resulting from a significant change in quadrupole moment upon photoexcitation.^1,2^ Such property is highly desirable for a broad range of applications, including *e.g.* two-photon fluorescence imaging,^3–6^ and two-photon initiated photopolymerisation.^7–9^ Donor–acceptor–donor systems have also been introduced as promising chromophores for thermally activated delayed fluorescence providing low singlet–triplet gaps along with suitable emission strengths.^10,11^ Additionally, these multi-branched dyes can be viewed as simple models of organic conjugated polymers and are particularly useful for understanding the electronic excited-state properties of these materials. Directly after optical excitation, the Franck–Condon excited state of these multi-branched dyes has a symmetric electronic structure with the excitation delocalized over the whole molecule. However, in many cases, excited-state symmetry breaking (ES-SB) occurs and leads to an uneven distribution of the excitation in the different branches and, in some cases, to a full localisation on a single branch. Over the last few years, the dynamics of ES-SB as well as the factors that influence this process have been intensively studied both experimentally,^12–32^ and theoretically.^33–38^ Theoretical investigations by Terenziani and Painelli suggested that ES-SB is triggered by solvent and structural fluctuations.^13,33,34^ Experimental studies revealed that the dynamics of ES-SB matches that of solvent motion.^16–20^ Inertial solvent motion is sufficient to break the symmetry on a sub-picosecond timescale. Further asymmetry is then achieved on a slower timescale *via* diffusive solvent motion (Fig. 1). Structural disorder or photochemical transformation were shown to be insufficient for ES-SB to take place in non-polar environments.^39,40^

Control on the extent of ES-SB can be achieved by tuning the properties of the local environment of the molecule with solvents of different polarity and H-bonding ability,^16–20,25,41^ as well as the strength of D and A subunits.^30^ The latter property is directly linked to the change of quadrupole moment upon excitation, which is also an important tuning parameter for ES-SB. Finally, the electronic coupling between the D–A branches is also a crucial factor for the extent of asymmetry.^35^ In strongly coupled systems, full localisation of the excitation on one branch is not possible even in the most polar environments.^18^ From a material point of view, ES-SB can be viewed as the decoherence of a delocalised multipolar exciton and its trapping in a smaller area. Consequently, the knowledge acquired upon investigating of ES-SB in multipolar molecules is also relevant to the understanding of the excited-state properties of conjugated materials.

**Fig. 2:**
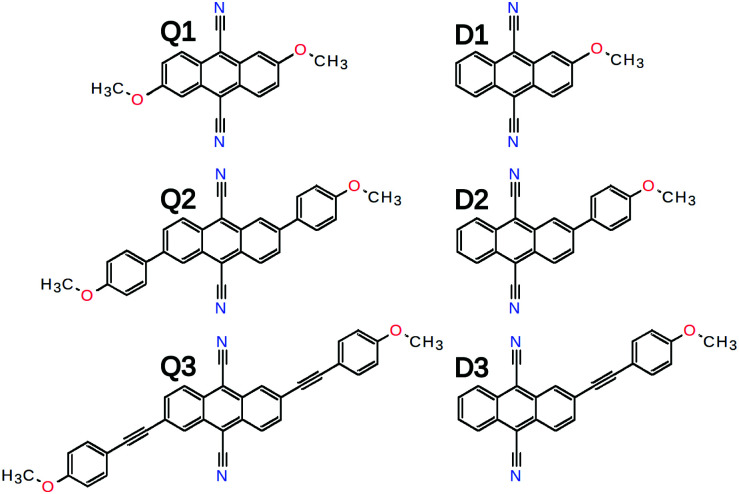
Chemical structure of the quadupolar D–A–D dyes and of their dipolar D–A analogues.

Here, we investigate the effect of the D–A branch length on ES-SB in a series of quadrupolar D–A–D dyes (Fig. 2). They consist of a 9,10-dicyanoanthracene (DCA) acceptor core substituted in 2 and 6 positions with two Ds that vary from methoxy (**Q1**) to 4-methoxyphenyl (**Q2**) and (4-methoxyphenyl)ethynyl (**Q3**) groups. The excited-state properties of these molecules are investigated by time-resolved vibrational spectroscopy in the mid-IR and electronic transient absorption in the Vis-NIR region and compared with those of the single-branch D–A analogues (**D1–3**) as well as those of the DCA core, which is itself a quadrupolar molecule. Time-resolved IR (TRIR) detection of ES-SB is usually done by probing vibrational modes localised on the D–A branches. This approach is only possible here with **Q3** and the –C≡C– stretching mode. However, ES-SB can also be detected by monitoring the appearance of Laporte-forbidden transitions in the transient absorption electronic spectrum.^42^ TRIR is also used here to probe the –C≡N stretching modes of the DCA sub-units and to understand how the symmetry of the core itself is affected by the presence of one or two D substituents. Our results reveal that the electronic distribution of the DCA core is very sensitive to the environment and to the donor. Moreover, a strong dependence of the extent of ES-SB on the branch length is observed and is discussed in terms of inter-branch coupling and solvation energy.

## 2 Results

The synthesis and basic photophysics of **Q1–3** and **D1–3** were discussed earlier.^43^ In brief, the absorption spectra of both **Q** and **D** series show no or minor solvatochromism, as expected for centro-symmetric molecules (**Q1–3**) and for molecules with a negligible permanent dipole moment in the ground state (**D1–3**). In contrast, their emission spectra exhibit a positive solvatochromism that increases with the length of donor–acceptor branch. For the D–A dyes, this can be simply explained by an excited-state dipole moment that increases with the branch length. For the D–A–D dyes, this points to a dipolar nature of the emissive state in polar solvents, hence to the occurrence of ES-SB. This interpretation is supported by the non-linear polarity dependence of the emission solvatochromism of **Q2** and **Q3**, that points to an excited-state dipole moment that increases with the solvent polarity.

It should be noted that DCA itself shows a weak but distinct fluorescence solvatochromism.^43^ Its origin is not totally clear. It can be explained without invoking ES-SB by a change of dipole–quadrupole interaction upon S_1_ → S_0_ transition. However, as discussed in ref. 40, this contribution to the solvatochromism is quantitatively not well described with the point quadrupole approximation. It should rather be viewed as a change of dipole–dipole interaction energy between the solvent and each of the two local dipoles of DCA.

**Fig. 3:**
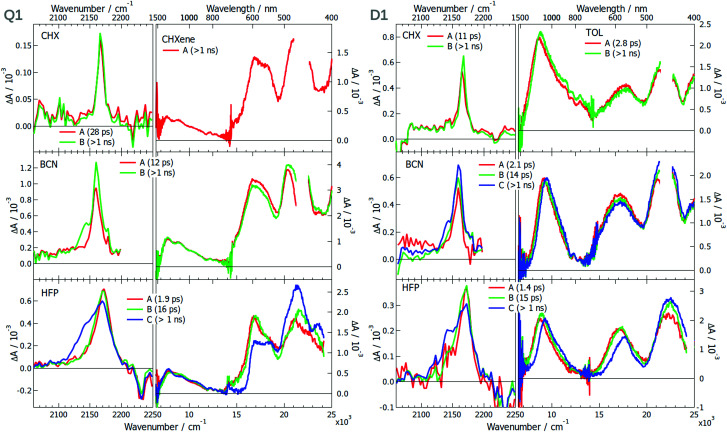
Evolution-associated difference spectra and time constants obtained from a global analysis of the TRIR and electronic TA spectra measured with **Q1** (left) and **D1** (right) in various solvents assuming a series of successive exponential steps. For BCN and HFP, the analysis was performed on the merged TRIR and electronic TA spectra. (CHX: cyclohexane; CHXene: cyclohexene; TOL: toluene; BCN: benzonitrile; HFP: hexafluoro-2-propanol).

Whereas TRIR measurements proved to be the workhorse for monitoring ES-SB dynamics,^16–19,25,30,40,41^ electronic transient absorption (TA) spectroscopy was less successful. However, we recently demonstrated that ES-SB can be visualised by monitoring the appearance of excited-state absorption (ESA) bands that are Laporte forbidden in the symmetric quadrupolar state.^42^ Therefore, the excited-state dynamics of all 6 dyes and DCA were investigated using both TRIR and TA spectroscopies in solvents of varying polarity. The transient data (TRIR-TA) collected in the same solvent were merged, and analysed globally assuming a series of successive exponential steps. The resulting evolution-associated difference spectra (EADS) are presented in Fig. 3, 5 and 6 and S8, S16, S30 (ESI†), whereas the original transient spectra are shown in Fig. S1–S7, S9–S15 and S23–S29 (ESI†).

The TRIR spectra measured in the triple bond stretching region with **Q1** in the apolar cyclohexane (CHX) exhibit a single ESA band at ∼2165 cm^−1^, which can be attributed to the antisymmetric –C≡N stretching mode (Fig. 3). As the electronic structure of **Q1** is expected to be centrosymmetric in non-polar solvents, the symmetric stretching mode is not IR active. Apart from a small increase that can be attributed to the equilibration of the S_1_ state, the ESA band decays on the nanosecond time scale, in agreement with the excited-state lifetime. This behaviour is very similar to that found with DCA in CHX (Fig. S8, ESI†). Although **D1** is not centrosymmetric, its TRIR spectra in CHX show a single ESA band at ∼2165 cm^−1^ as well. However, time-dependent density functional theory (TD-DFT) calculations of **D1** in the S_1_ state predict a 7 cm^−1^ frequency difference between the symmetric and the antisymmetric –C≡N stretching modes and an oscillator strength smaller by a factor 6.3 for the former. The ESA band due to the symmetric –C≡N vibration could, thus, possibly be hidden by the more intense band of the antisymmetric mode. Because of the limited solubility of **Q1** in CHX, the electronic TA measurements were performed in cyclohexene (CHXene), which can also be considered as a non-polar solvent. The TA spectra exhibit two intense ESA bands above 15 000 cm^−1^ and a weaker one around 7500 cm^−1^. No significant dynamics can be observed apart that associated with the decay of the S_1_ state on the ns time scale. These spectra resemble those measured with DCA in CHX (Fig. S8, ESI†). The TA spectra measured with **D1** in the low-polar toluene are very similar as well, except for the low-energy ESA, which is at 9000 cm^−1^ and is the most intense band. Weak spectral dynamics, that is probably due to solvent relaxation,^44^ can also be observed at early time.

The strong difference in the intensity of the lowest-energy ESA band between **Q1** and **D1** is consistent with the lower symmetry of **D1**. Indeed, the Laporte rule for absorption holds for centrosymmetric molecules like **Q1**, but is no longer valid for molecules lacking a centre of inversion like **D1**.^45^ Based on this, we propose that the intense 9000 cm^−1^ band of **D1** is due to a transition that is forbidden for the centrosymmetric **Q1**. The band at 7500 cm^−1^ is either due to this forbidden transition, hence its weaker intensity, or to another transition, that is also present in the spectrum of **D1** but hidden under the intense 9000 cm^−1^ band.

The TRIR spectra measured at early time with **Q1** in the polar benzonitrile (BCN) and hexafluoro-2-propanol (HFP) are identical to those in CHX. However, a second ESA band, appearing as a shoulder, rises within a few ps at ∼2140 cm^−1^. It shifts to lower frequencies and becomes more intense upon going from BCN to the strongly protic HFP. This band can be assigned to the symmetric –C≡N stretching mode and its appearance points to a loss of symmetry of the two –C≡N groups. Such a behaviour is also present with DCA, but only in HFP, and is less pronounced (Fig. S30, ESI†). Only a single ESA band is observed in the polar but aprotic acetonitrile (Fig. S16, ESI†).

Surprisingly, the TRIR spectra measured with **D1** exhibit a very similar solvent dependence as those of **Q1**, although this molecule is intrinsically asymmetric. This behaviour could be explained by a charge-transfer character of the S_1_ state of **D1** and a difference of electronic density on the two cyano groups that both increase by going from CHX to BCN and HFP. Although HFP is less polar than BCN, it is highly protic,^46^ and can form H-bonds with the –C≡N nitrogen, significantly stabilising a larger charge-transfer character of the excited state.^17^ The second ESA band rises on a similar time scale as that of solvent relaxation, in agreement with this hypothesis.

The fact that **Q1** and **D1** exhibit the same TRIR spectra points to a similar asymmetry in the electronic density on the cyano groups. Interestingly, such asymmetry is also present with DCA, but only in HFP, where it is enabled by the H-bond interactions. However, these TRIR results do not tell whether the electronic density on the two donors is symmetric or not.

The electronic TA spectra measured with **Q1** and **D1** in BCN and HFP are essentially the same as those in CHX. The only difference is some early spectral dynamics, more visible in HFP, that can be associated with solvent relaxation (Tables S2–S8, ESI†).^17,30,44^ The decay of the ESA band of **Q1** at ∼17 000 cm^−1^ and the rise of that at ∼22 000 cm^−1^ can be accounted for by the dynamic Stokes shift of the stimulated emission (Fig. S17, ESI†). As in CHX, the intensity of the lowest-energy electronic ESA band in polar solvents is significantly smaller for **Q1** than **D1** and does not exhibit any significant dynamics. According to the above hypothesis based on the Laporte rule, this points to a similar electronic density on the two D groups of **Q1** even in polar solvents. Solute–solvent interactions are sufficient to slightly break the symmetry of –C≡N groups but too weak to make the electronic structure of the S_1_ state of **Q1** similar to that of **D1**. Our results show that the presence of one (**D1**) or two D groups (**Q1**) facilitates the loss of symmetry of the two –C≡N groups, most probably by increasing the electronic density on the DCA core.

The –C≡N stretching frequency is known to be highly sensitive to electronic density.^47–49^ The 20–30 cm^−1^ splitting of the –C≡N bands found here in HFP is very modest compared the 130 cm^−1^ band splitting observed with another A–D–A type quadupolar molecule in the same solvent.^17,18^

**Fig. 4:**
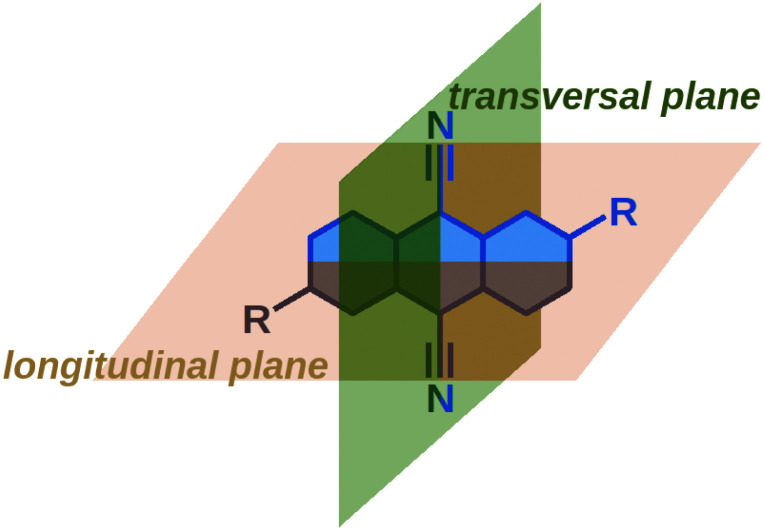
Designation of the longitudinal and transversal planes of 9,10-dicyanoanthracenes.

These results reveal that the TRIR and electronic TA spectra report on the symmetry of the electronic distribution relatively to different molecular planes of the dye. The –C≡N stretching band intensities in the TRIR spectra are sensitive to the symmetry with respect to the longitudinal plane (Fig. 4). As the S_1_ ← S_0_ transition of these dyes is significantly affected by the donor substituents, the Laporte rule in the electronic TA spectra reflects mainly the symmetry of the electronic distribution along the long D–A–D molecular axis, *i.e.* with respect to the transversal plane. The electronic TA spectra suggest that **Q1** still preserves a symmetry relatively to this transversal plane.

**Fig. 5:**
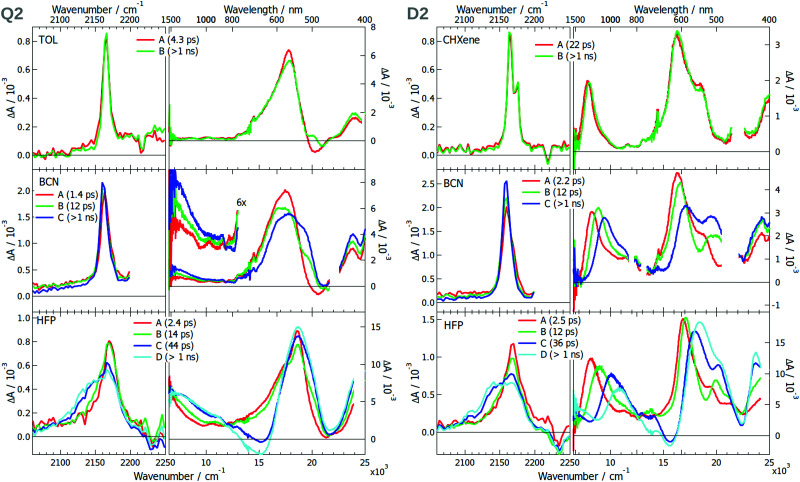
Evolution-associated difference spectra and time constants obtained from a global analysis of the merged TRIR and electronic TA spectra measured with **Q2** (left) and **D2** (right) in various solvents assuming a series of successive exponential steps.(CHXene: cyclohexene; TOL: toluene; BCN: benzonitrile; HFP: hexafluoro-2-propanol).

The EADS obtained after global analysis of the TRIR and electronic TA data obtained with **Q2** and **D2** are presented in Fig. 5. The TRIR spectra of **Q2** in TOL exhibit a single ESA band, which decays on the ns time scale. This contrasts with the spectrum measured with **D2** in CHXene, which exhibit two ESA bands split by only 10 cm^−1^. This points to a symmetry in the electronic density on the –C≡N groups with **Q2** but not with **D2**. The electronic TA spectra measured with **Q2** present a single ESA band around 17 000 cm^−1^ whereas those measured with **D2** show additionally an ESA band at 8000 cm^−1^. The absence of this band with **Q2** can be explained by a Laporte-forbidden transition. The TRIR spectra measured with **Q2** and **D2** in the polar BCN exhibit a single ESA band at ∼2165 cm^−1^ (Fig. 5). In HFP, a second band rises at 2140 cm^−1^ with a 12–14 ps time constant. The absence of a second band in BCN is surprising but can be explained by frequency down-shift of the symmetric –C≡N band by going from TOL to BCN and HFP (see Section S2.3, ESI† for a discussion on the solvent dependence of the band splitting). Consequently, this band shifts from the high-frequency side of the antisymmetric –C≡N band in low-polar solvents to the other side in the highly protic HFP, and overlaps with it in BCN. The larger width of the ESA band of **Q2** in BCN compared to TOL agrees with the presence of two weakly split bands and, thus, suggests asymmetry of the two –C≡N groups. Such different sensitivities of the symmetric and antisymmetric –C≡N stretching frequencies to the electronic density was reported previously.^49^

The electronic TA data evidence ES-SB in two D–A branches of **Q2** in polar solvents. At early time, the spectrum measured in BCN is similar to that in toluene with a single ESA band. However, a second band rises around 7500 cm^−1^ on a similar time scale as that of solvent motion. Such a rise is also occurring in HFP, although the band is already visible in the early spectra. The appearance of this band, present in the spectrum of **D2**, is a clear indication of ES-SB and originates from the loss of the centre of inversion of the excited state wavefunction.^42^ The presence of this band in the early spectra in HFP can be explained by ES-SB occurring through inertial solvent motion already.

The spectral dynamics around 17 000 cm^−1^ visible for both **Q2** and **D2** can be attributed to the shift of the stimulated emission band due to solvent relaxation (Fig. S19–S20, ESI†). The associated time constants agree with the typical relaxation times of these solvents.^17,44^

**Fig. 6:**
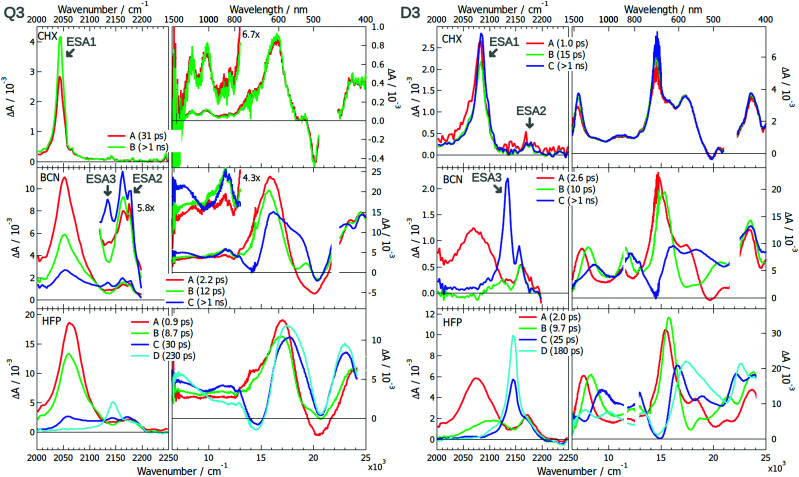
Evolution-associated difference spectra and time constants obtained from a global analysis of the merged TRIR and electronic TA spectra measured with **Q3** (left) and **D3** (right) in various solvents assuming a series of successive exponential steps.(CHX: cyclohexane; BCN: benzonitrile; HFP: hexafluoro-2-propanol).

The excited-state dynamics of **Q3** and **D3** was already partially discussed.^42^ The EADS obtained from the global analysis of the TRIR and electronic data measured with these dyes in various solvents are presented in Fig. 6. In brief, the TRIR spectra of both compounds are dominated by a single ESA band (ESA1) in CHX that was assigned to the –C≡C– stretching mode. The ESA band due the antisymmetric –C≡N mode, ESA2, is much weaker and hardly visible with **Q3**. Only the antisymmetric –C≡C– stretching mode is active with **Q3**, pointing to a symmetric and quadrupolar S_1_ state. The intensity in the –C≡N region is too weak to draw conclusions on the DCA core. The electronic TA spectra measured with **Q3** exhibit a relatively weak band at ∼9000 cm^−1^ with a vibronic structure. Based on quantum chemical calculations, this band was assigned to the symmetry-allowed S_3_ ← S_1_ transition.^42^ These calculations also predicted Laporte-forbidden S_2_ ← S_1_ and S_4_ ← S_1_ transitions at 6170 and 10 900 cm^−1^, respectively. Comparatively, the TA spectra measured with **D3** show an intense ESA band around 7000 cm^−1^, that most probably corresponds to the S_2_ ← S_1_ transition.

Significant spectral dynamics is observed in the TRIR and TA spectra measured with **Q3** and **D3** in polar solvents. The most important ones visible in the TRIR spectra of **Q3** are (i) the splitting of ESA2, pointing to a loss of symmetry relatively to the longitudinal plane, and (ii) the decrease of –C≡C– ESA1 and the concurrent increase of a new band, ESA3, around 2145 cm^−1^, that was assigned, on the basis of anisotropy measurements, to a –C≡C– stretching vibration. Very similar TRIR spectra are observed with **D3**, the main difference being the smaller relative intensity of ESA1, in agreement with the presence of a single –C≡C– bond. The decay of ESA1 and the rise of ESA3 in **D3** was attributed to the equilibration of the Franck–Condon S_1_ state, which involves an increase of the charge-transfer character as solvent relaxation takes place, in agreement with the non-linear fluorescence solvatochromism observed with **D3**. The comparable spectral dynamics found with **Q3** also point to an increase of the charge-transfer character of the S_1_ state. The identical position of ESA3 for **Q3** and **D3** indicates a –C≡C– bond with very similar electronic density, hence a dipolar excited state for both molecules. One can thus conclude that ES-SB is taking place with **Q3**.

Unambiguous evidence of ES-SB in **Q3** is given by the TA spectra. Whereas they are similar to those in CHX at early time, two bands rise around 7000 and 11 500 cm^−1^ within a few tens of ps. According to quantum-chemical calculations, they can be assigned to the above-mentioned Laporte-forbidden S_2_ ← S_1_ and S_4_ ← S_1_ transitions, that become allowed upon ES-SB.^42^ By contrast, no new rising bands are observed with **D3**, the only dynamics being associated with spectral shifts on the time scale of solvent relaxation (Fig. S21 and S22, ESI†). The time constants obtained from global analysis suggest solvent-controlled excited-state dynamics for both dyes: relaxation to a state with enhanced charge-transfer character with **D3** and ES-SB with **Q3**.

## 3 Discussion

The spectroscopic results can be summarised as follows: the electronic structure of all three excited D–A–D dyes remains symmetric in non-polar and weakly polar solvents. In polar solvents, the data reveal a correlation between propensity of the initially quadrupolar excited state to undergo symmetry breaking and the D–A branch length. In **Q1**, the electron donor is directly linked to the DCA core, resulting in the shortest branch length of all dyes. The strong similarity of the transient spectra of **Q1** and DCA points to a modest effect of the methoxy donors on the symmetry of the electronic structure. Apart from a relatively small asymmetry of the cyano groups, that is also present in DCA but to a smaller extent, the excited state of **Q1** remains mostly symmetric even in highly polar solvents. The donor branches (4-methoxyphenyl groups) of **Q2** are longer and the electronic distribution of its S_1_ state exhibit a higher sensitivity to the polarity of the environment. In BCN, the initially symmetry-forbidden S_2_ ← S_1_ transition becomes allowed as solvation energy favours an uneven distribution of the excitation on the two D–A branches, a so-called 'intermediate' state (Fig. 1). In the highly protic HFP, the intensity of the S_2_ ← S_1_ band is similar to that observed with **D2**, pointing to a more dipolar character of the excited state. **Q3** is characterised by the longest D–A branches as well as by the highest sensitivity of its excited state to the polarity of the environment. In BCN, the Laporte-forbidden transitions become visible in the electronic TA spectra upon solvent relaxation, indicating an electronic structure practically identical to that observed with **D3** in polar solvents.

**Fig. 7:**
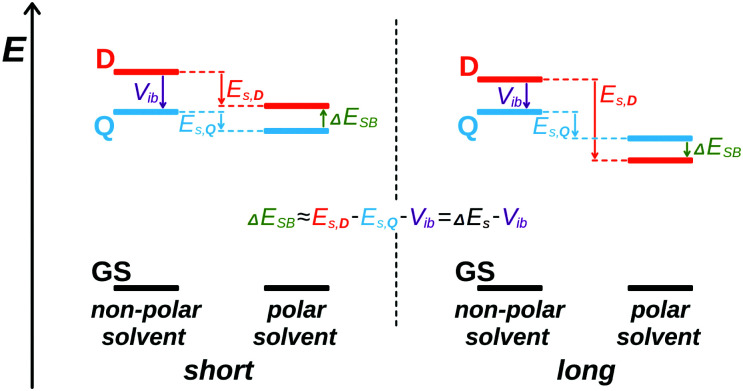
Schematic illustration of the influence of branch length on the relative magnitude of the excitonic interaction and solvation energy and on the relative energies of the symmetric (Q) and symmetry-broken (D) excited states.

This influence of the D–A branch length on the tendency to undergo ES-SB can be understood by considering a simple model where the D and A groups are identical and only their separation differs. This D–A distance, *d*_DA_, affects both the interbranch coupling energy, *V*_ib_, and the dipolar solvation energy, *E*_s_. The former favours a symmetric distribution of the excitation over both branches, whereas the latter stabilises a dipolar state with the excitation localised on a single D–A branch. Therefore, the energy difference between the dipolar symmetry-broken state, and the symmetric quadrupolar excited state, Δ*E*_SB_, can be approximated to (Fig. 7):

1 Δ*E*_SB_ = *E*_D_ − *E*_Q_ ≈ *E*_s,D_ − *E*_s,Q_ − *V*_ib_ = Δ*E*_s_ − *V*_ib_,

where *E*_D_, *E*_Q_, *E*_s,D_ and *E*_s,Q_ are the energy and the dipolar solvation energy of the symmetry-broken and of the symmetric excited states, respectively, and Δ*E*_s_ is the change of solvation energy upon ES-SB.

The inter-branch coupling, *V*_ib_, can be estimated from the Kasha's excitonic model,^50^ assuming that each D–A branch is an individual chromophore with a transition dipole of magnitude *M*_DA_:2
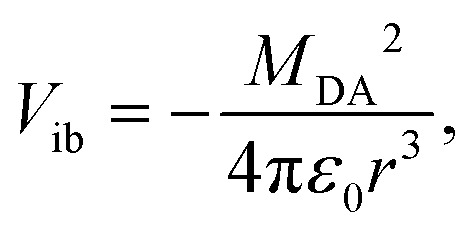
where *r* is the distance between the transition dipoles. In the point dipole approximation, *r* = *d*_DA_. The magnitude of the transition dipole, *M*_DA_, also depends on the length of the D–A branch. For a pure charge-transfer (CT) transition, it is given by:^51–53^3
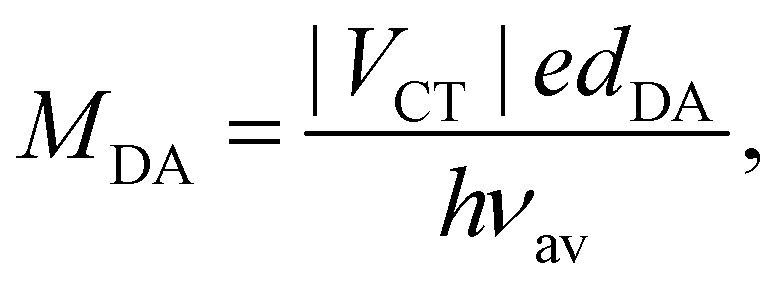
where *V*_CT_ is the electronic coupling between ground and the CT state and *ν*_av_ is the average absorption frequency. Therefore, in the limit of pure CT transition, the branch length dependence of the inter-branch coupling is:4
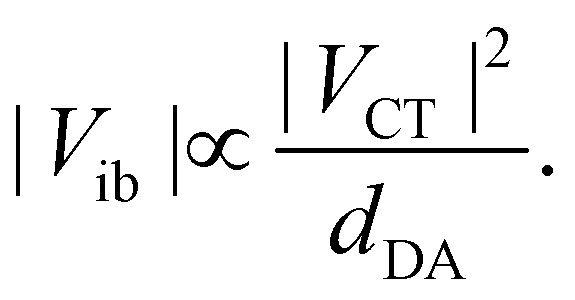


The coupling *V*_CT_ is closely related to the electronic coupling between the A and D subunits and is generally assumed to decay exponentially with the D–A distance.^54,55^ Consequently, increasing the branch length when going from **Q1** to **Q3** is predicted to be accompanied by a significant diminution of *V*_ib_.

For centrosymmetric molecules like **Q1–3**, |*V*_ib_| corresponds to half the energy difference between the first two-photon allowed transition and the first one-photon allowed transition from the ground state.^12^ In the Kasha's model, this energy difference corresponds to the Davidov excitonic splitting.^50^ The |*V*_ib_| values estimated from the one- and two-photon absorption spectra reported in ref. 43 are listed in Table 1. They confirm a decrease of the inter-branch coupling by going from **Q1** to **Q3**. According to quantum-chemical calculations, the first one- and two- photon absorption bands correspond to the S_1_ ← S_0_ and S_3_ ← S_0_ transitions, respectively.^42,43^Table 1 reveals that, although the *V*_ib_ values deduced from these calculated excitation energies are larger than those determined from the measured spectra, they also decrease with increasing the D–A branch length.

**Table 1:** Estimated inter-branch coupling, *V*_ib_, and solvation energies of the dipolar state in BCN, *E*_s,D_(BCN), expressed in cm^−1^. *ν̃*_TPA_: wavenumber of the first two-photon allowed absorption band; *ν̃*_OPA_: wavenumber of the first one-photon allowed absorption band; *E*_S1_ and *E*_S3_: energies of the first and third excited states taken from ref. 43

	**Q1**	**Q2**	**Q3**
|*V*_ib_| ≈ (*ν̃*_TPA_ − *ν̃*_OPA_)/2	3600	3000	2200
|*V*_ib_| ≈ (*E*_S3_ − *E*_S1_)/2	4700	3000	2700
−*E*_s,D_(BCN)	775	2260	3275

On the other hand, the increase of solvation energy upon ES-SB can be estimated within the dielectric continuum model and the point dipole approximation (Section S3, ESI†):^56^5
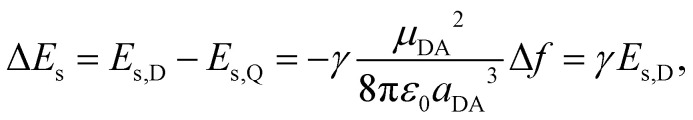
where *μ*_DA_ is the magnitude of dipole moment of the dipolar symmetry-broken state, *a*_DA_ is the cavity radius of one D–A branch, *γ* is a factor ranging from 1/2 to 7/8 depending on the method used to estimate the cavity radius of the quadrupolar state (Section S3, ESI†), and Δ*f* = *f*(*ε*) − *f*(*n*^2^) with the static dielectric constant *ε*, the refractive index *n* and the Onsager function *f*(*x*) = 2(*x* − 1)/(2*x* + 1).

As *μ*_DA_ = *δed*_DA_, where *δ* is the amount of charge transfer in the excited state, and *a*_DA_ = *d*_DA_/2, the gain of solvation energy upon ES-SB is predicted to vary with the branch length as:6
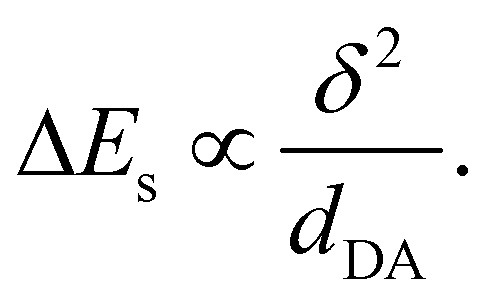


The amount of charge transfer, *δ*, can be related the electronic coupling, *V*_CT_:^53^7
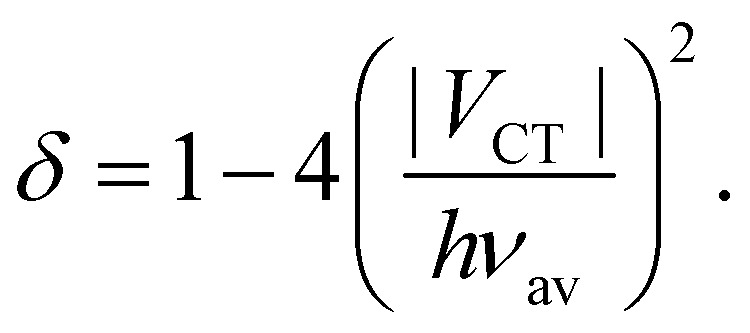


Indeed, full charge transfer, *δ* = 1, requires the D and A subunits to be totally decoupled.

Comparison of eqn (4) and (6) indicates that both *V*_ib_ and Δ*E*_s_ scale with *d*_DA_^−1^. However, their dependence on *V*_CT_ are opposite: *V*_ib_ increases with *V*_CT_, whereas Δ*E*_s_ decreases. As *V*_CT_ decays exponentially with *d*_DA_, a lengthening the D–A branches should result in a diminution of *V*_ib_, as observed (Table 1), and in an augmentation of Δ*E*_s_.

The estimated solvation energies of the dipolar excited state of **Q1–3** in BCN, *E*_s,D_(BCN), are listed in Table 1. They were calculated using the results of the solvatochromism study reported in ref.43 by taking half of the shift of the fluorescence band of **D1–3** upon changing the dielectric constant from the CHX to the BCN value. This table points to a substantial increase of the gain of solvation energy upon ES-SB when going from **Q1** to **Q3**. This is in good agreement with the predictions based on eqn (5)–(7).

The estimated inter-branch coupling and solvation energies listed in Table 1 agree well with the experimental results. For **Q1**, the gain of solvation energy in polar solvents upon ES-SB is clearly too small to compensate for the loss of excitonic stabilisation. Therefore, the excited state of **Q1** is predicted to be symmetric even in the polar BCN, as observed (Fig. 7, left). For **Q2**, *V*_ib_ and Δ*E*_s_ in BCN are of similar order of magnitude. This is also consistent with the results that point to a partial localisation of the excitation in BCN (intermediate state). Finally, for **Q3**, the gain of solvation energy in BCN can compete with the inter-branch coupling, pointing to a symmetry-broken electronic structure in this solvent, as found experimentally (Fig. 7, right).

These results reveal a clear correlation between the propensity of quadrupolar D–A–D molecules to undergo ES-SB and the length of the D–A branches. According to the above simple model, this dependence can be traced to the electronic coupling, *V*_CT_, which decreases exponentially with the D–A branch length. A decrease in *V*_CT_ results on the one hand in a decrease of the transition dipole moment, *M*_DA_, hence of the inter-branch coupling, and, on the other hand, in an increase of excited-state dipole moment of a single branch, *μ*_DA_, and thus of the solvation energy.

## 4 Conclusions

Excited-state symmetry breaking was investigated in quadrupolar D–A–D dyes with a 9,10-dicyanoanthracene acceptor core and methoxy donors, allowing for an investigation of the influence of the D–A branch length on the propensity to undergo ES-SB. The latter was monitored using both time-resolved infrared spectroscopy in the triple bond stretching region and electronic transient absorption spectroscopy in the Vis-NIR region. ES-SB in these dyes was only found in polar solvents with the medium and longest D–A branches. With the shortest branches, the excited state was found to remain symmetric even in highly polar solvents. These results were rationalised using a simple model where, for ES-SB to be operative, the loss of excitonic interaction, or inter-branch coupling, must be compensated by a gain in solvation energy. Excitonic interaction and solvation energy show an opposite dependence on the D–A branch length, the former decreasing with increasing length and the latter increasing. These dependences can be attributed to the electronic coupling between the D and A subunits, which decays exponentially with distance.

The branch length dependence found here should be quite general for multipolar dyes. Additionally to the branch length, further tuning can also be achieved by variation of the D and A strength. For a given branch length, a stronger donor should result in a smaller electronic coupling between D and A, and, thus, in a smaller inter-branch coupling. As a consequence, ES-SB in polar solvents could occur already with short D–A branches. These results should facilitate the design of new dyes where ES-SB is desired or not, depending on the foreseen application.

## Conflicts of interest

There are no conflicts to declare.

## Supplementary Material

CP-023-D1CP02376D-s001
