# Supplementary material for: Excited-state symmetry breaking in 9,10-dicyanoanthracene-based quadrupolar molecules: the effect of donor–acceptor branch length
Source: Phys Chem Chem Phys. 2021 Jul 2;23(28):15150–8. doi: 10.1039/d1cp02376d (PMC8294646; doi:10.1039/d1cp02376d)
Supplement: CP-023-D1CP02376D-s001 [file CP-023-D1CP02376D-s001.pdf]

Electronic Supplementary Material (ESI) for Physical Chemistry Chemical Physics.
This journal is © the Owner Societies 2021

Electronic Supporting Information

—

Effect of donor-acceptor branch length on excited-state symmetry breaking in 9,10-dicyanoanthracene-based quadrupolar molecules

Zoltán Szakács,[1] Florian Glöcklhofer,[2] Felix Plasser,[3] Eric Vauthey[1]*

[1] *Department of Physical Chemistry, University of Geneva, 30 Quai Ernest-Ansermet, 1211 Genève 4, Switzerland*

[2] *Department of Chemistry and Centre for Processable Electronics, Imperial College London, Molecular Sciences Research Hub, 80 Wood Lane, London, W12 0BZ, UK*

[3] *Department of Chemistry Loughborough University, Loughborough, LE11 3TU, UK*

Contents

S1 Materials and Methods **S4**
S1.1 Materials S4
S1.1.1 Solvent properties S4
S1.2 Spectroscopic methods S4
S1.2.1 Stationary measurements S4
S1.2.2 Time-resolved infrared spectroscopy S4
S1.2.3 Electronic transient absorption spectroscopy S5
S1.3 Quantum-chemical calculations S5

S2 Results **S6**
S2.1 Global analysis S6
S2.2 TRIR and electronic TA spectra S8
S2.2.1 In non-polar/low-polarity solvents S8
S2.2.2 In polar solvents S10
S2.2.3 In hexafluoro-2-propanol S15
S2.3 Polarity dependent down-shift of the symmetric -C≡N stretching mode in **D2** S18

S3 Solvation energy of a quadrupole vs. a dipole **S19**

List of Figures

S1 TRIR and electronic TA spectra measured with **Q1**, in cyclohexane (TRIR), in cyclohexene (electronic TA) S8

S2 TRIR and electronic TA spectra measured with **D1**, in cyclohexane (TRIR), in toluene (electronic TA) . . . S8
S3 TRIR and electronic TA spectra measured with **Q2** in toluene . . . S8
S4 TRIR and electronic TA spectra measured with **D2** in cyclohexene . . . S9
S5 TRIR and electronic TA spectra measured with **Q3** in cyclohexane . . . S9
S6 TRIR and electronic TA spectra measured with **D3** in cyclohexane . . . S9
S7 TRIR and electronic TA spectra measured with **DCA** in cyclohexane . . . S10
S8 Evolution-associated difference spectra and time constants obtained from a global analysis of the merged TRIR and electronic TA spectra measured with **DCA** in cyclohexane . . . S10
S9 TRIR and electronic TA spectra measured with **Q1** in benzonitrile . . . S10
S10 TRIR and electronic TA spectra measured with **D1** in benzonitrile . . . S11
S11 TRIR and electronic TA spectra measured with **Q2** in benzonitrile . . . S11
S12 TRIR and electronic TA spectra measured with **D2** in benzonitrile . . . S11
S13 TRIR and electronic TA spectra measured with **Q3** in benzonitrile . . . S12
S14 TRIR and electronic TA spectra measured with **D3** in benzonitrile . . . S12
S15 TRIR and electronic TA spectra measured with **DCA** in acetonitrile . . . S12
S16 Evolution-associated difference spectra and time constants obtained from a global analysis of the merged TRIR and electronic TA spectra measured with **DCA** in acetonitrile . . . S13
S17 Evolution associated difference spectra obtained from the TRIR and electronic TA spectra measured with **Q1** in benzonitrile, the stationary emission spectra measured in CHX and in BCN are plotted in black and grey, respectively . . . S13
S18 Evolution associated difference spectra obtained from the TRIR and electronic TA spectra measured with **D1** in benzonitrile, the stationary emission spectra measured in CHX and in BCN are plotted in black and grey, respectively . . . S13
S19 Evolution associated difference spectra obtained from the TRIR and electronic TA spectra measured with **Q2** in benzonitrile, the stationary emission spectra measured in CHX and in BCN are plotted in black and grey, respectively . . . S14
S20 Evolution associated difference spectra obtained from the TRIR and electronic TA spectra measured with **D2** in benzonitrile, the stationary emission spectra measured in CHX and in BCN are plotted in black and grey, respectively . . . S14
S21 Evolution associated difference spectra obtained from the TRIR and electronic TA spectra measured with **Q3** in benzonitrile, the stationary emission spectra measured in CHX and in BCN are plotted in black and grey, respectively . . . S14
S22 Evolution associated difference spectra obtained from the TRIR and electronic TA spectra measured with **D3** in benzonitrile, the stationary emission spectra measured in CHX and in BCN are plotted in black and grey, respectively . . . S15
S23 TRIR and electronic TA spectra measured with **Q1** in hexafluoro-2-propanol . . . S15
S24 TRIR and electronic TA spectra measured with **D1** in hexafluoro-2-propanol . . . S15
S25 TRIR and electronic TA spectra measured with **Q2** in hexafluoro-2-propanol . . . S16
S26 TRIR and electronic TA spectra measured with **D2** in hexafluoro-2-propanol . . . S16
S27 TRIR and electronic TA spectra measured with **Q3** in hexafluoro-2-propanol . . . S16
S28 TRIR and electronic TA spectra measured with **D3** in hexafluoro-2-propanol . . . S17
S29 TRIR and electronic TA spectra measured with **DCA** in hexafluoro-2-propanol . . . S17

S30 Evolution-associated difference spectra and time constants obtained from a global analysis of the merged TRIR and electronic TA spectra measured with **DCA** in hexafluoro-2-propanol . . . S17
S31 TRIR spectra measured with **D2** in cyclohexene, dibutyl ether, benzonitrile and N-methylformamide . . . S18
S32 Definition of the cavity radius for the quadupolar state (A, B) and the dipolar state (C) . . . S19

S1 Materials and Methods

S1.1 Materials

The synthesis of **Q1**, **Q2**, **Q3**, **D1**, **D2** and **D3** was described in Ref. 1. Cyclohexane (CHX, ROTISOLV HPLC, >99.9 %) was obtained from Carl Roth Gmbh. Toluene (TOL, Extra Dry over Molecular Sieve, >99.85 %) and dibutyl ether (DBE, Extra Dry, AcroSeal, >99.0) were purchased from Acros Organics. Benzonitrile (BCN, CHROMASOLV HPLC, >99.9 %) and 1,1,1,3,3,3-hexafluoro-2-propanol (HFP, >99.0 %) were purchased from Sigma-Aldrich. N-methylformamide (NMF, >99 %) and cyclohexene (CHXene, laboratory reagent grade) were obtained from Fischer Chemicals AG. DCA was purchased from Kodak.

S1.1.1 Solvent properties

Table S1: Solvent properties (n: refractive index; ϵ : static dielectric constant; $f(\epsilon)-f(n^2)$: Onsager polarity function, with $f(x) = 2(x-1)/(2x+1)$.

solvent	n	ϵ	$f(\epsilon) - f(n^2)$
cyclohexane	1.43	2.0	0.00
cyclohexene	1.45	2.22	0.02
toluene	1.50	2.38	0.02
dibutyl ether	1.40	3.1	0.19
benzonitrile	1.53	25.2	0.47
N-methylformamide	1.43	182.4	0.58
acetonitrile	1.34	37.5	0.61
hexafluoro-2-propanol	1.28	16.7	0.61

S1.2 Spectroscopic methods

S1.2.1 Stationary measurements

Electronic absorption spectra were recorded on a Cary 50 spectrometer. Stationary fluorescence spectra were measured using a Horiba FluoroMax-4 spectrofluorometer and corrected using a set of secondary emissive standards.[2] Stationary IR absorption spectra were recorded with a Bio-Rad Excalibur FTIR spectrometer in a NaCl cell using THF as solvent.

S1.2.2 Time-resolved infrared spectroscopy

Time-resolved infrared (TRIR) spectroscopy measurements were performed using a setup described in detail elsewhere.[3, 4] Briefly, the pump pulses at 450 nm were generated by doubling the 900 nm output of an optical parametric amplifier (TOPAS C, Light Conversion) pumped by a Ti-Sapphire amplified laser system (Spectra-Physics, Solstice amplifier, 1 kHz). The pump pulses at 400 nm were produced by doubling the 800 nm output of the same amplifier. The IR probe pulses were generated from another part of the output of the same laser system using an optical parametric amplifier (TOPAS C, Light Conversion) and a non-collinear difference frequency mixing module (NDFG, Light Conversion). The polarisations of the pump and probe pulses were controlled and

the pump pulses were set at magic angle relative to the probe. The probe pulses were dispersed in a Triax 190 spectrograph (Horiba, 150 lines/mm) and detected with an MCT array (2 × 64 pixels, Infrared Sytems Development). All TRIR data were measured using a flow-through cell made of CaF_2 windows with a 500 μm spacer. The absorbance of the samples at 450 nm was less than 0.3 in the cell.

S1.2.3 Electronic transient absorption spectroscopy

- **UV-Vis transient absorption spectroscopy**

  UV-Vis transient absorption spectra were recorded on a setup described in detail elsewhere.[5, 6] In brief, the pump pulses at 460 nm were generated using a TOPAS-Prime combined with a frequency mixer (Light Conversion) and the TOPAS was pumped by a Ti-Sapphire amplified system (Spectra-Physics, Solstice Ace amplifier, 1 kHz). The pump pulses at 400 nm were generated by doubling the 800 nm output of the same amplifier. The white light continuum probe pulses were produced by focusing the 800 nm pulses of the amplifier in a CaF_2 plate. The polarization of both, pump and probe pulses, was controlled and the pump was set at magic angle relative to the probe. The signal was recorded applying a referenced detection system of two spectrographs. Pixel to wavelength conversion was performed using a NIST 2065 standard. The measured data were chirp-corrected using the optical Kerr effect measured in the neat solvent samples. All the samples were measured in 1 mm quartz cuvettes and bubbled with N_2. A wavelength dependent instrument response function (IRF) was observed and an IRF of 200-350 fs was achieved. The absorbance of the sample at the pump wavelength was less then 0.3 in the 1 mm quartz cuvette.

- **Near-IR transient absorption spectroscopy**

  Near-IR transient absorption spectroscopy was performed using a setup similar to the UV-Vis transient absorption setup and the same amplifier was used to generate the pump and probe pulses. The main differences are as follows, all the samples were excited at 400 nm. The probe pulse were generated by focusing the 800 nm output of the amplifier in a YAG plate. The detection was done by a referenced home-built prism spectrometer consisting of two InGaAs detectors. For balancing the white light continuum spectrum, apodizing neutral density filters were placed front of both detectors.[6]

- **Merging the UV-Vis and Near-IR spectra**

  In order to reconstruct the electronic transient absorption spectra from 6500 cm^{-1} to 30000 cm^{-1}, the chirp corrected UV-Vis and near-IR spectra were then merged by comparing the overlapping ~710-770 nm spectral region. The near-IR spectra were multiplied by a constant factor for correcting for the difference of pump power, as well as the differences of sample absorbance at the pump wavelength.

S1.3 Quantum-chemical calculations

Quantum-chemical calculations were performed using the Gaussian16 package.[7] The ground-state and excited-state geometries were optimised without symmetry restriction at the density functional theory (DFT) level and time-dependent DFT (TD-DFT) level using the ωB97X-D long-range corrected hybrid density functional,[8] with the standard 6-31+G* basis set in vacuum. Frequency

calculations were performed on the optimised structures at the DFT level and no imaginary frequencies were found, indicating that the observed stationary points are real minima of the potential energy surface (PES).

S2 Results

S2.1 Global analysis

Global analysis was performed on the merged electronic TA spectra and TRIR spectra assuming a series of consecutive exponential steps with increasing time constants. The resulting evolution associated difference spectra (EADS) are shown in the main text figures.

Table S2: Time constants obtained from the global analysis of spectra measured with **Q1** in various solvents, TA: electronic TA spectra, TRIR: TRIR spectra and TA & TRIR: merged TA & TRIR spectra were used in the global analysis

		**Q1**		
	dataset	τ_1 (ps)	τ_2 (ps)	τ_3 (ps)
cyclohexane	TRIR	28	>1000	-
cyclohexene	TA	>1000	-	-
benzonitrile	TA & TRIR	12	>1000	-
hexafluoro-2-propanol	TA & TRIR	1.9	16	>1000

Table S3: Time constants obtained from the global analysis of spectra measured with **D1** in various solvents, TA: electronic TA spectra, TRIR: TRIR spectra and TA & TRIR: merged TA & TRIR spectra were used in the global analysis

		**D1**		
	dataset	τ_1 (ps)	τ_2 (ps)	τ_3 (ps)
cyclohexane	TRIR	11	>1000	-
toluene	TA	2.8	>1000	-
benzonitrile	TA & TRIR	2.1	14	>1000
hexafluoro-2-propanol	TA & TRIR	1.4	15	>1000

Table S4: Time constants obtained from the global analysis of spectra measured with **Q2** in various solvents, TA & TRIR: merged TA & TRIR spectra were used in the global analysis

		**Q2**			
	dataset	τ_1 (ps)	τ_2 (ps)	τ_3 (ps)	τ_4 (ps)
toluene	TA & TRIR	4.3	>1000	-	-
benzonitrile	TA & TRIR	1.4	12	>1000	-
hexafluoro-2-propanol	TA & TRIR	2.4	14	44	>1000

Table S5: Time constants obtained from the global analysis of spectra measured with **D2** in various solvents, TA & TRIR: merged TA & TRIR spectra were used in the global analysis

		**D2**			
	dataset	τ_1 (ps)	τ_2 (ps)	τ_3 (ps)	τ_4 (ps)
cyclohexene	TA & TRIR	22	>1000	-	-
benzonitrile	TA & TRIR	2.2	12	>1000	-
hexafluoro-2-propanol	TA & TRIR	2.5	12	36	>1000

Table S6: Time constants obtained from the global analysis of spectra measured with **Q3** in various solvents, TA & TRIR: merged TA & TRIR spectra were used in the global analysis

		**Q3**			
	dataset	τ_1 (ps)	τ_2 (ps)	τ_3 (ps)	τ_4 (ps)
cyclohexane	TA & TRIR	31	>1000	-	-
benzonitrile	TA & TRIR	2.2	12	>1000	-
hexafluoro-2-propanol	TA & TRIR	0.9	8.7	30	230

Table S7: Time constants obtained from the global analysis of spectra measured with **D3** in various solvents, TA & TRIR: merged TA & TRIR spectra were used in the global analysis

		**D3**			
	dataset	τ_1 (ps)	τ_2 (ps)	τ_3 (ps)	τ_4 (ps)
cyclohexane	TA & TRIR	1.1	15	>1000	-
benzonitrile	TA & TRIR	2.6	10	>1000	-
hexafluoro-2-propanol	TA & TRIR	2.0	9.7	25	180

Table S8: Time constants obtained from the global analysis of spectra measured with **DCA** in various solvents, TA & TRIR: merged TA & TRIR spectra were used in the global analysis

		**DCA**			
	dataset	τ_1 (ps)	τ_2 (ps)	τ_3 (ps)	τ_4 (ps)
cyclohexane	TA & TRIR	2.2	>1000	-	-
acetonitrile	TA & TRIR	1.7	48	>1000	-
hexafluoro-2-propanol	TA & TRIR	1.3	17	99	>1000

S2.2 TRIR and electronic TA spectra

S2.2.1 In non-polar/low-polarity solvents

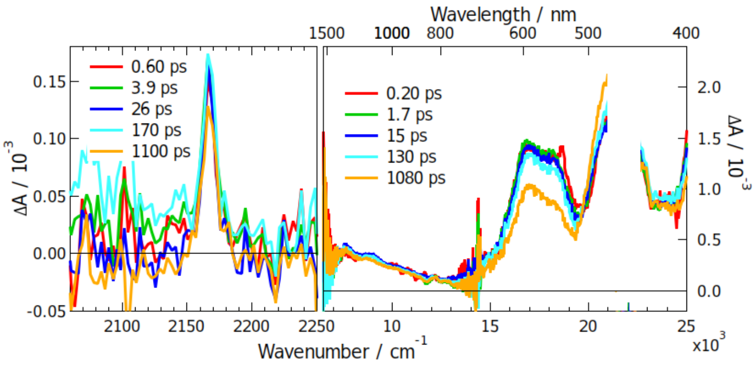

Figure S1: TRIR and electronic TA spectra measured with **Q1**, in cyclohexane (TRIR), in cyclohexene (electronic TA)

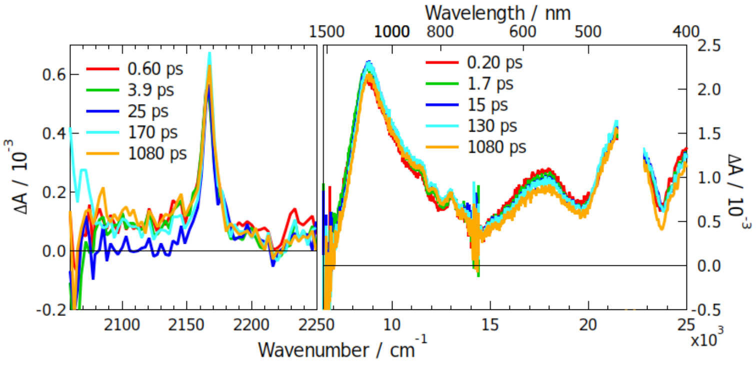

Figure S2: TRIR and electronic TA spectra measured with **D1**, in cyclohexane (TRIR), in toluene (electronic TA)

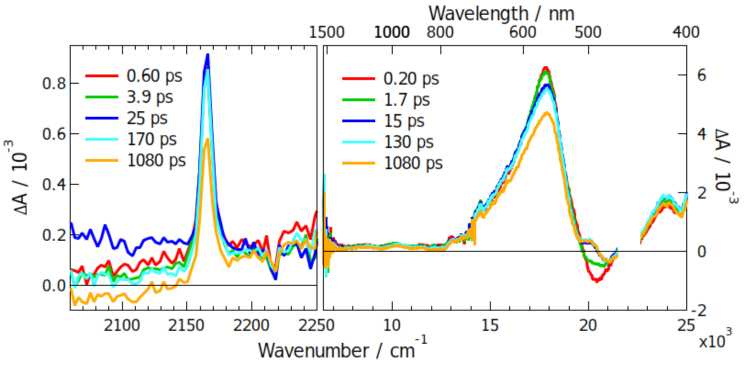

Figure S3: TRIR and electronic TA spectra measured with **Q2** in toluene

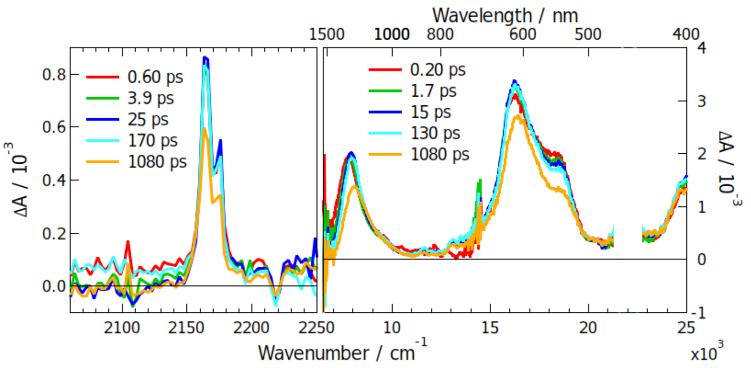

Figure S4: TRIR and electronic TA spectra measured with **D2** in cyclohexene

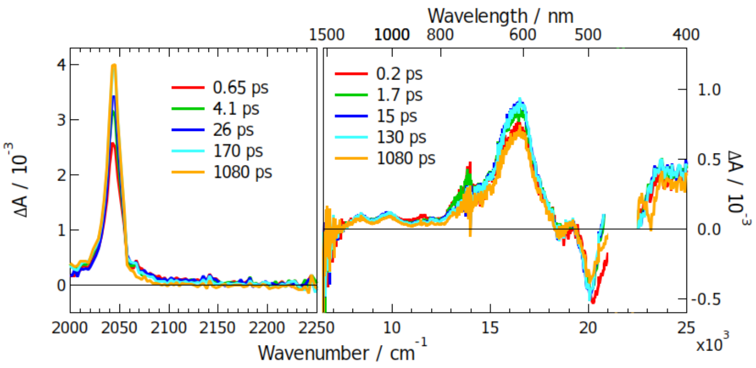

Figure S5: TRIR and electronic TA spectra measured with **Q3** in cyclohexane

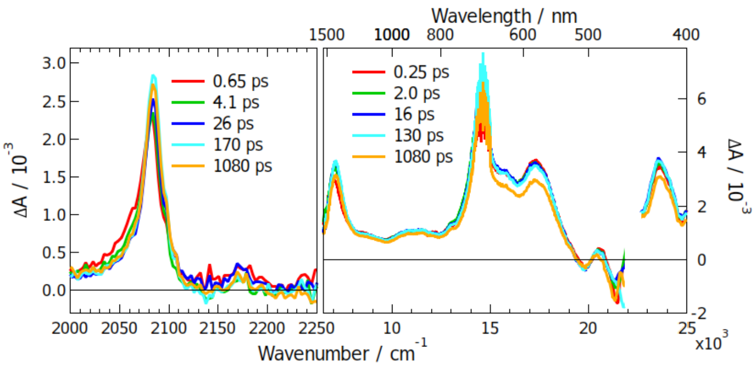

Figure S6: TRIR and electronic TA spectra measured with **D3** in cyclohexane

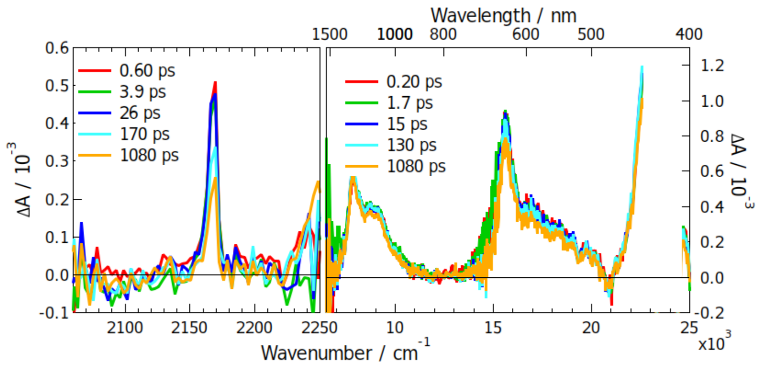

Figure S7: TRIR and electronic TA spectra measured with **DCA** in cyclohexane

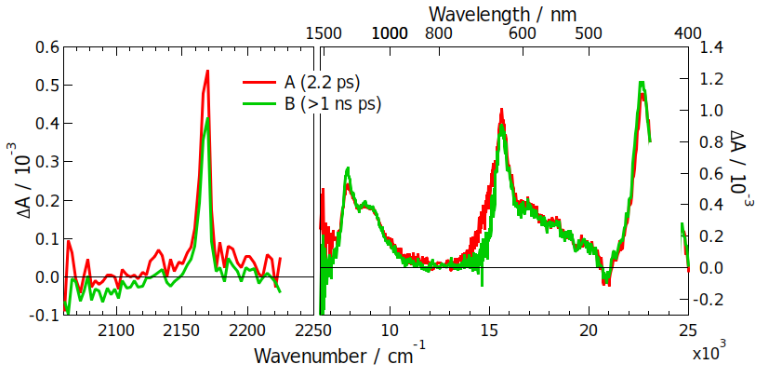

Figure S8: Evolution-associated difference spectra and time constants obtained from a global analysis of the merged TRIR and electronic TA spectra measured with **DCA** in cyclohexane

S2.2.2 In polar solvents

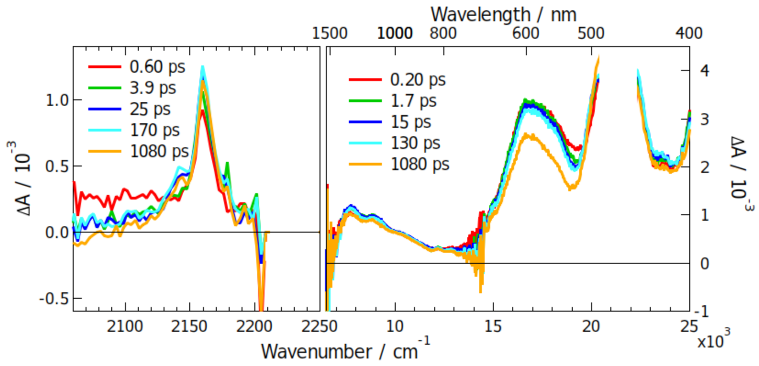

Figure S9: TRIR and electronic TA spectra measured with **Q1** in benzonitrile

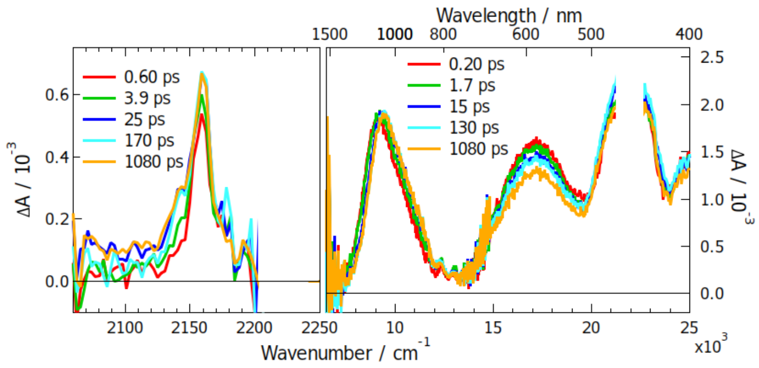

Figure S10: TRIR and electronic TA spectra measured with **D1** in benzonitrile

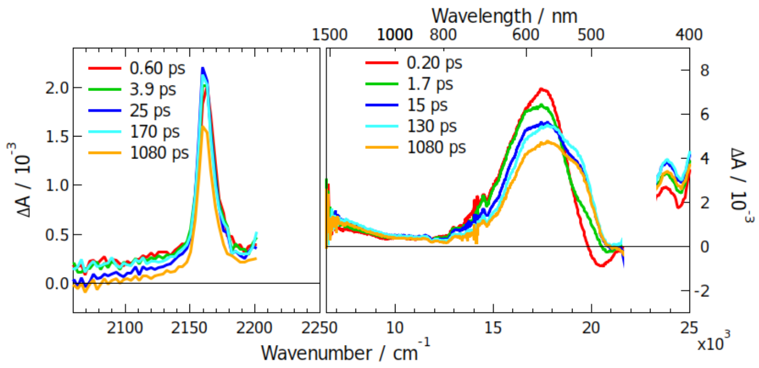

Figure S11: TRIR and electronic TA spectra measured with **Q2** in benzonitrile

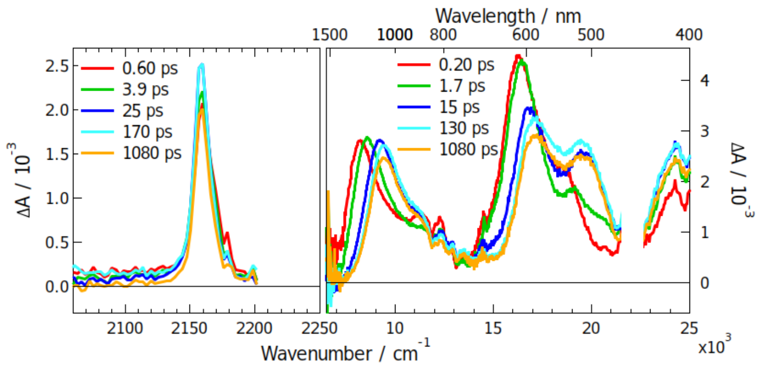

Figure S12: TRIR and electronic TA spectra measured with **D2** in benzonitrile

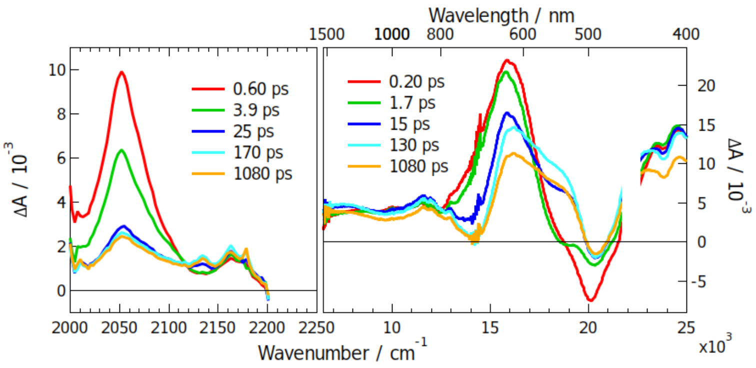

Figure S13: TRIR and electronic TA spectra measured with **Q3** in benzonitrile

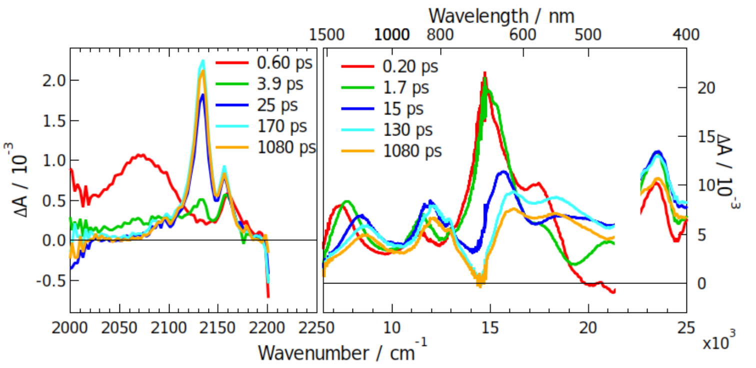

Figure S14: TRIR and electronic TA spectra measured with **D3** in benzonitrile

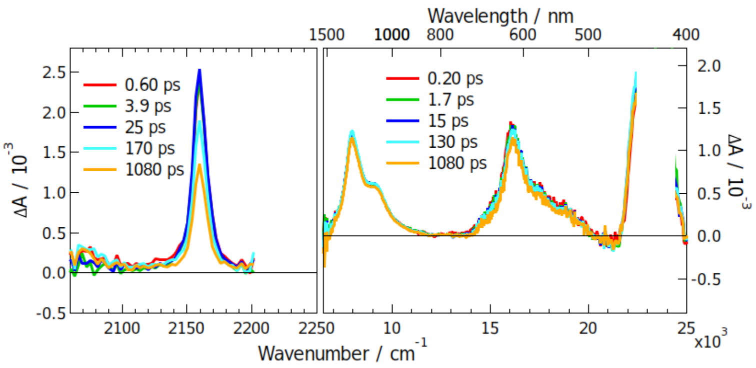

Figure S15: TRIR and electronic TA spectra measured with **DCA** in acetonitrile

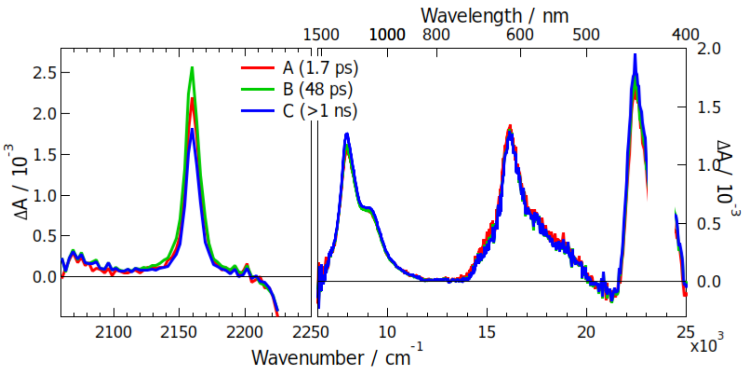

Figure S16: Evolution-associated difference spectra and time constants obtained from a global analysis of the merged TRIR and electronic TA spectra measured with **DCA** in acetonitrile

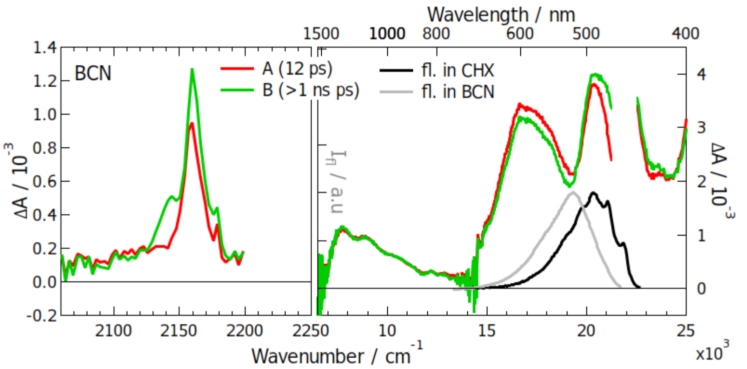

Figure S17: Evolution associated difference spectra obtained from the TRIR and electronic TA spectra measured with **Q1** in benzonitrile, the stationary emission spectra measured in CHX and in BCN are plotted in black and grey, respectively

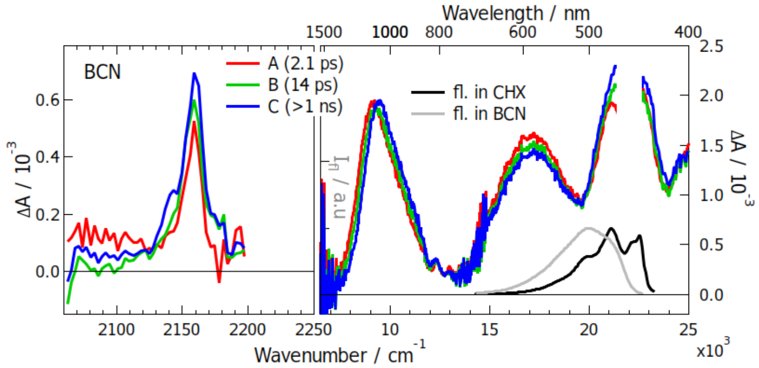

Figure S18: Evolution associated difference spectra obtained from the TRIR and electronic TA spectra measured with **D1** in benzonitrile, the stationary emission spectra measured in CHX and in BCN are plotted in black and grey, respectively

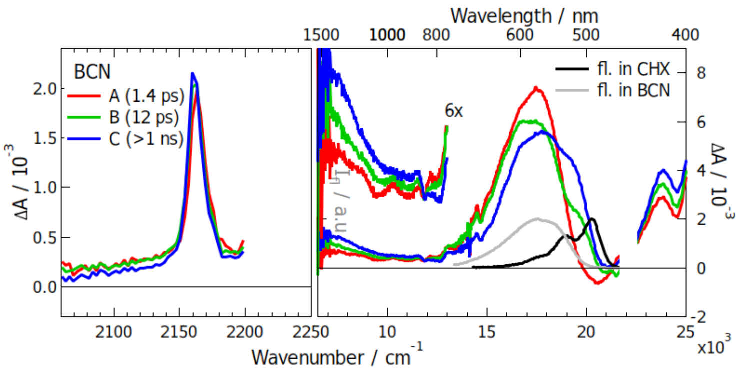

Figure S19: Evolution associated difference spectra obtained from the TRIR and electronic TA spectra measured with **Q2** in benzonitrile, the stationary emission spectra measured in CHX and in BCN are plotted in black and grey, respectively

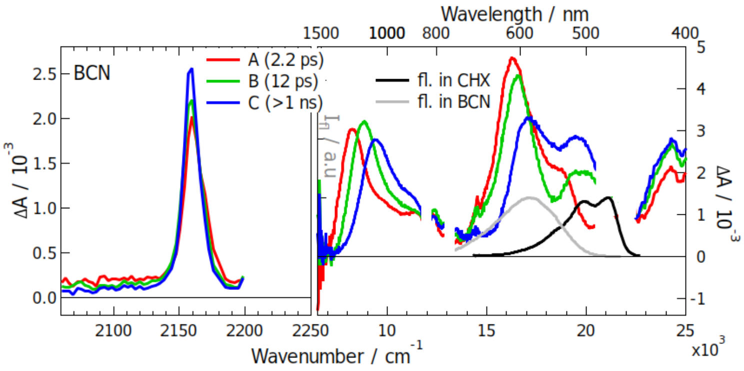

Figure S20: Evolution associated difference spectra obtained from the TRIR and electronic TA spectra measured with **D2** in benzonitrile, the stationary emission spectra measured in CHX and in BCN are plotted in black and grey, respectively

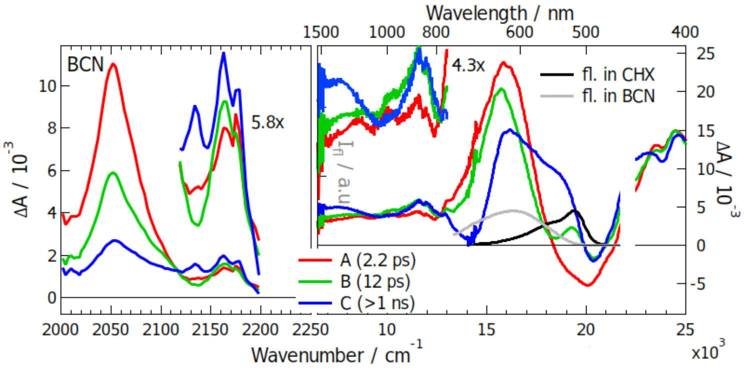

Figure S21: Evolution associated difference spectra obtained from the TRIR and electronic TA spectra measured with **Q3** in benzonitrile, the stationary emission spectra measured in CHX and in BCN are plotted in black and grey, respectively

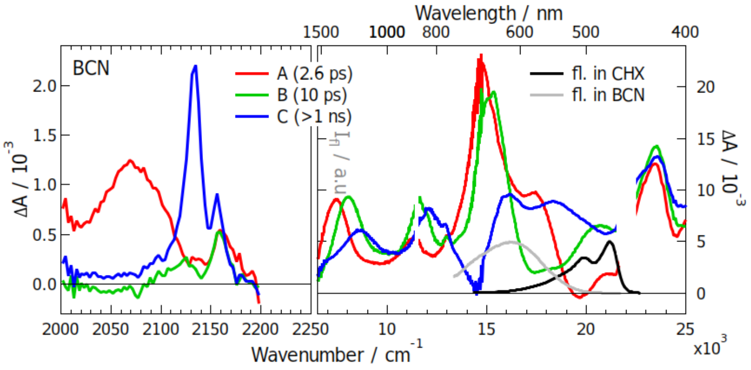

Figure S22: Evolution associated difference spectra obtained from the TRIR and electronic TA spectra measured with **D3** in benzonitrile, the stationary emission spectra measured in CHX and in BCN are plotted in black and grey, respectively

S2.2.3 In hexafluoro-2-propanol

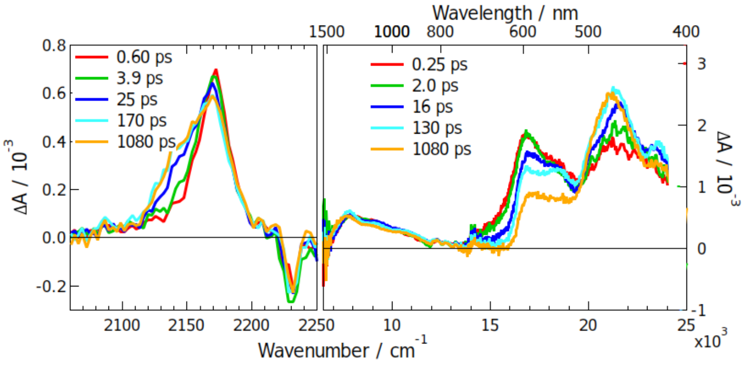

Figure S23: TRIR and electronic TA spectra measured with **Q1** in hexafluoro-2-propanol

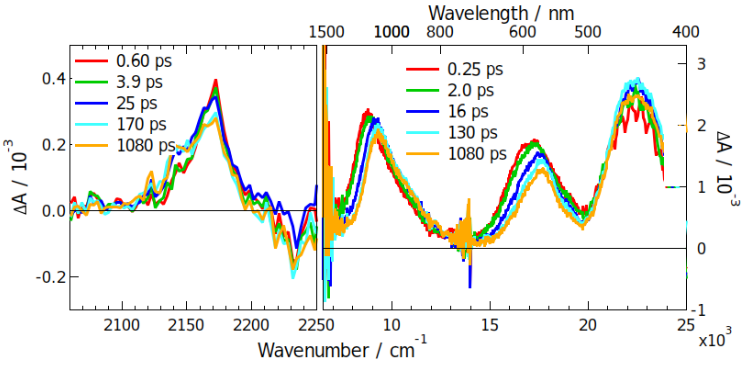

Figure S24: TRIR and electronic TA spectra measured with **D1** in hexafluoro-2-propanol

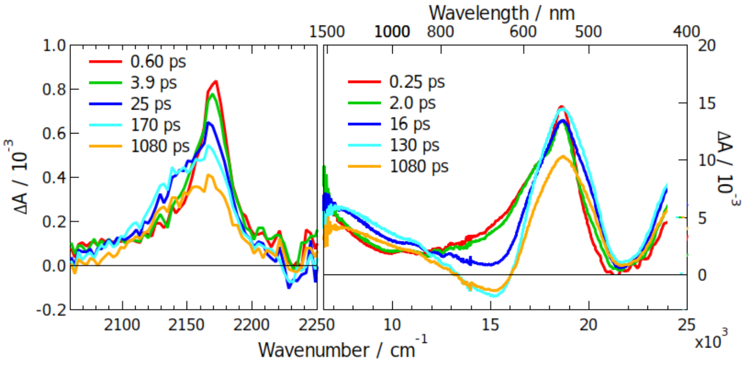

Figure S25: TRIR and electronic TA spectra measured with **Q2** in hexafluoro-2-propanol

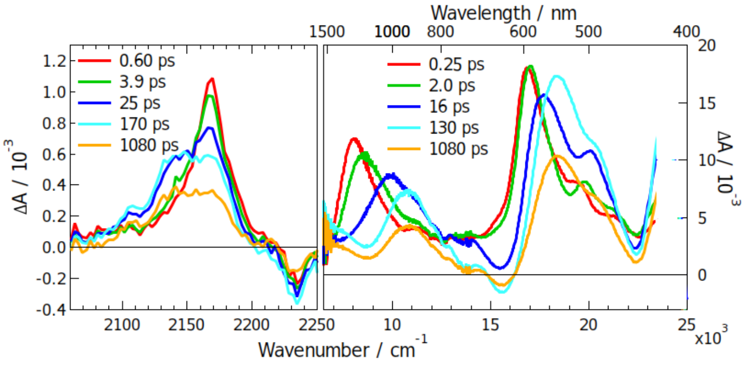

Figure S26: TRIR and electronic TA spectra measured with **D2** in hexafluoro-2-propanol

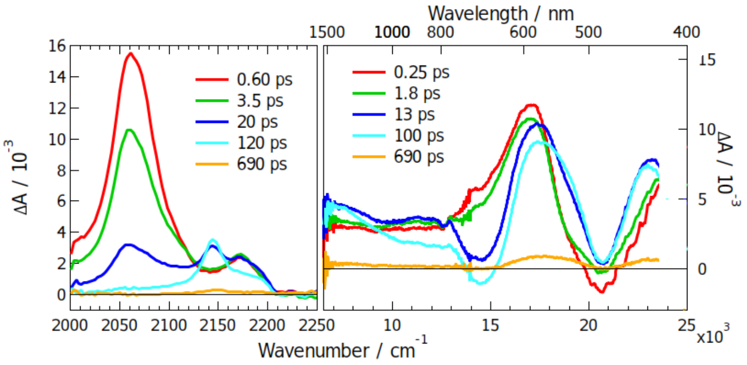

Figure S27: TRIR and electronic TA spectra measured with **Q3** in hexafluoro-2-propanol

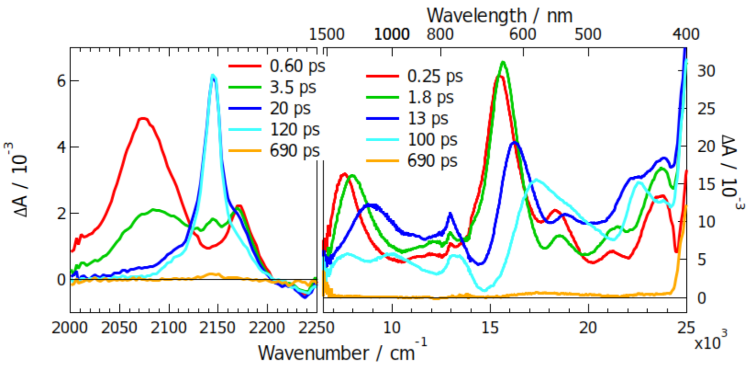

Figure S28: TRIR and electronic TA spectra measured with **D3** in hexafluoro-2-propanol

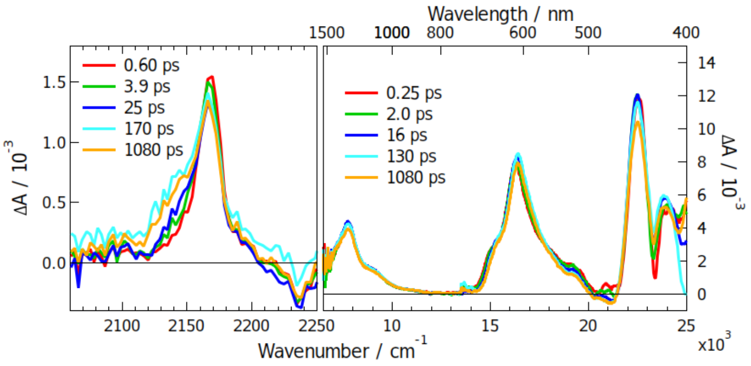

Figure S29: TRIR and electronic TA spectra measured with **DCA** in hexafluoro-2-propanol

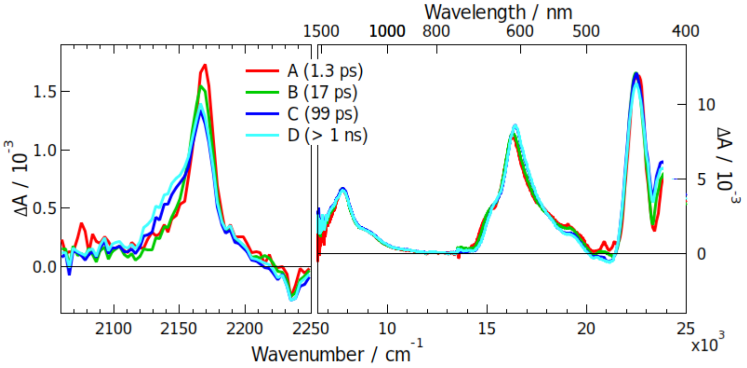

Figure S30: Evolution-associated difference spectra and time constants obtained from a global analysis of the merged TRIR and electronic TA spectra measured with **DCA** in hexafluoro-2-propanol

S2.3 Polarity dependent down-shift of the symmetric -C≡N stretching mode in D2

The absence of the symmetric -C≡N stretching mode in **D2** in BCN was investigated by measuring the TRIR spectra in solvents of varying polarity. The resulting spectra are shown in Figure S31. They point to a polarity dependent redshift of the symmetric stretching mode. When measuring in DBE, the symmetric -C≡N stretching mode red-shifts by $\sim 10\,\mathrm{cm}^{-1}$ compared to the spectra obtained in CHXene, while in BCN and NMF, it is further red-shifts and completely overlaps with the more intense antisymmetric mode.

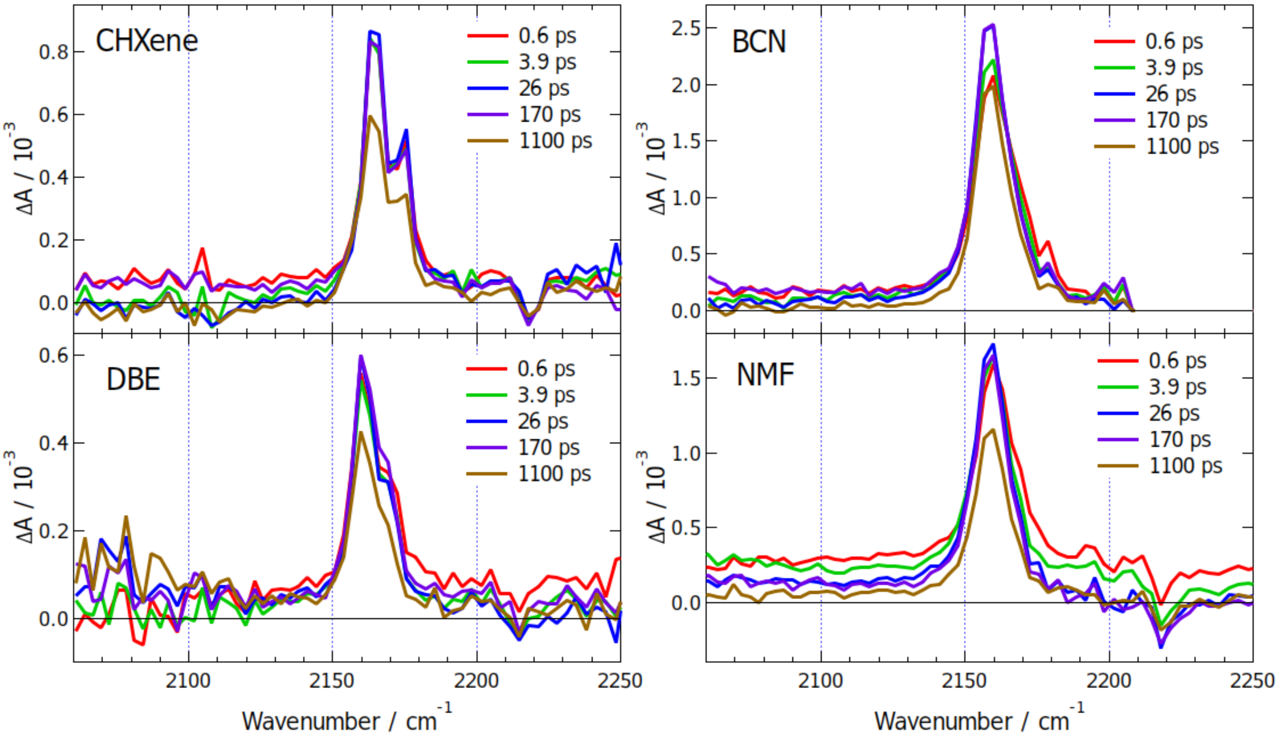

Figure S31: TRIR spectra measured with **D2** in cyclohexene, dibutyl ether, benzonitrile and N-methylformamide

S3 Solvation energy of a quadrupole vs. a dipole

The magnitude of a quadupole moment, Q, consisting of two local dipoles of magnitude $\mu/2$ is (Figure S32A):

$$Q = \mu d, \tag{S1}$$

where d is the centre-to-centre distance between the two (point) local dipoles.

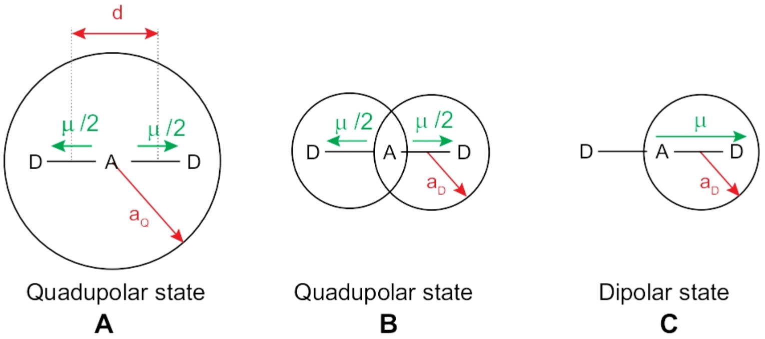

Figure S32: Definition of the cavity radius for the quadupolar state (A, B) and the dipolar state (C)

According to the continuum model, the dipolar solvation energy of a quadrupole is:[9]

$$E_{\mathrm{s,Q}} = -\frac{Q^2}{8\pi\epsilon_0 a_{\mathrm{Q}}^5}\Delta f = -\frac{\mu^2 d^2}{8\pi\epsilon_0 a_{\mathrm{Q}}^5}\Delta f, \tag{S2}$$

where a_{Q} is the cavity radius (Figure 1A), and $\Delta f = f(\epsilon_s) - f(n^2)$, with $f(x) = 2(x-1)/(2x+1)$.

With the assumption that $d \sim a_{\mathrm{Q}}$, eq.S2 becomes:

$$E_{\mathrm{s,Q}} \simeq -\frac{\mu^2}{8\pi\epsilon_0 a_{\mathrm{Q}}^3}\Delta f. \tag{S3}$$

The dipolar solvation energy of a dipole of size μ in a cavity of radius a_{D} is (Figure S32C):[9]

$$E_{\mathrm{s,D}} = -\frac{\mu^2}{8\pi\epsilon_0 a_{\mathrm{D}}^3}\Delta f. \tag{S4}$$

Comparison of eq.S3 and S4 shows that, because the cavity radius of the quadrupole is larger than that of the dipole, the solvation energy of the dipolar state is larger than that of the quadrupolar state.

Assumption that $a_{\mathrm{Q}} \sim 2a_{\mathrm{D}}$ gives:

$$E_{\mathrm{s,Q}} \simeq -\frac{\mu^2}{64\pi\epsilon_0 a_{\mathrm{D}}^3}\Delta f = \frac{1}{8}E_{\mathrm{s,D}}. \tag{S5}$$

This approach probably underestimates $E_{\mathrm{s,Q}}$ because of the large cavity radius.

Alternatively, if the quadrupole is described as two local dipoles, each of radius a_{D} (Figure S32B), the solvation energy is:

$$E_{\mathrm{s,Q}} \simeq -2\frac{(\mu/2)^2}{8\pi\epsilon_0 a_{\mathrm{D}}^3}\Delta f = \frac{1}{2}E_{\mathrm{s,D}}. \tag{S6}$$

Here, the solvation energy is probably overestimated. Nevertheless, both eq.S5 and S6 indicate that dipolar solvation favours the purely dipolar state D rather than the symmetric quadrupolar state Q.

References

[1] F. Glöcklhofer, A. Rosspeintner, P. Pasitsuparoad, S. Eder, J. Fröhlich, G. Angulo, E. Vauthey and F. Plasser, *Mol. Syst. Des. Eng.*, 2019, **4**, 951–961.

[2] J. A. Gardecki and M. Maroncelli, *Appl. Spectrosc.*, 1998, **52**, 1179–1189.

[3] M. Koch, R. Letrun and E. Vauthey, *J. Am. Chem. Soc.*, 2014, **136**, 4066–4074.

[4] B. Dereka, *PhD thesis*, University of Geneva, 2018.

[5] N. Banerji, G. Duvanel, A. Perez-Velasco, S. Maity, N. Sakai, S. Matile and E. Vauthey, *J. Phys. Chem. A*, 2009, **113**, 8202–8212.

[6] A. Aster, G. Licari, F. Zinna, E. Brun, T. Kumpulainen, E. Tajkhorshid, J. Lacour and E. Vauthey, *Chem. Sci.*, 2019, **10**, 10629–10639.

[7] M. J. Frisch, G. W. Trucks, H. B. Schlegel, G. E. Scuseria, M. A. Robb, J. R. Cheeseman, G. Scalmani, V. Barone, G. A. Petersson, H. Nakatsuji, X. Li, M. Caricato, A. V. Marenich, J. Bloino, B. G. Janesko, R. Gomperts, B. Mennucci, H. P. Hratchian, J. V. Ortiz, A. F. Izmaylov, J. L. Sonnenberg, D. Williams-Young, F. Ding, F. Lipparini, F. Egidi, J. Goings, B. Peng, A. Petrone, T. Henderson, D. Ranasinghe, V. G. Zakrzewski, J. Gao, N. Rega, G. Zheng, W. Liang, M. Hada, M. Ehara, K. Toyota, R. Fukuda, J. Hasegawa, M. Ishida, T. Nakajima, Y. Honda, O. Kitao, H. Nakai, T. Vreven, K. Throssell, J. A. Montgomery, Jr., J. E. Peralta, F. Ogliaro, M. J. Bearpark, J. J. Heyd, E. N. Brothers, K. N. Kudin, V. N. Staroverov, T. A. Keith, R. Kobayashi, J. Normand, K. Raghavachari, A. P. Rendell, J. C. Burant, S. S. Iyengar, J. Tomasi, M. Cossi, J. M. Millam, M. Klene, C. Adamo, R. Cammi, J. W. Ochterski, R. L. Martin, K. Morokuma, O. Farkas, J. B. Foresman and D. J. Fox, *Gaussian-16 Revision C.01*, 2016, Gaussian Inc. Wallingford CT.

[8] J.-D. Chai and M. Head-Gordon, *Phys. Chem. Chem. Phys.*, 2008, **10**, 6615–6620.

[9] P. Suppan and N. Ghoneim, *Solvatochromism*, The Royal Society of Chemistry, Cambridge, 1997.
